# Omics Technology for the Promotion of Nutraceuticals and Functional Foods

**DOI:** 10.3389/fphys.2022.817247

**Published:** 2022-05-13

**Authors:** Deepu Pandita, Anu Pandita

**Affiliations:** ^1^ Government Department of School Education, Jammu, India; ^2^ Vatsalya Clinic, New Delhi, India

**Keywords:** functional food, nutraceuticals, nutrigenomics, transcriptomics, proteomics, metabolomics, epigenomics, miRNomics

## Abstract

The influence of nutrition and environment on human health has been known for ages. Phytonutrients (7,000 flavonoids and phenolic compounds; 600 carotenoids) and pro-health nutrients—nutraceuticals positively add to human health and may prevent disorders such as cancer, diabetes, obesity, cardiovascular diseases, and dementia. Plant-derived bioactive metabolites have acquired an imperative function in human diet and nutrition. Natural phytochemicals affect genome expression (nutrigenomics and transcriptomics) and signaling pathways and act as epigenetic modulators of the epigenome (nutri epigenomics). Transcriptomics, proteomics, epigenomics, miRNomics, and metabolomics are some of the main platforms of complete omics analyses, finding use in functional food and nutraceuticals. Now the recent advancement in the integrated omics approach, which is an amalgamation of multiple omics platforms, is practiced comprehensively to comprehend food functionality in food science.

## Introduction

**FIGURE 1:**
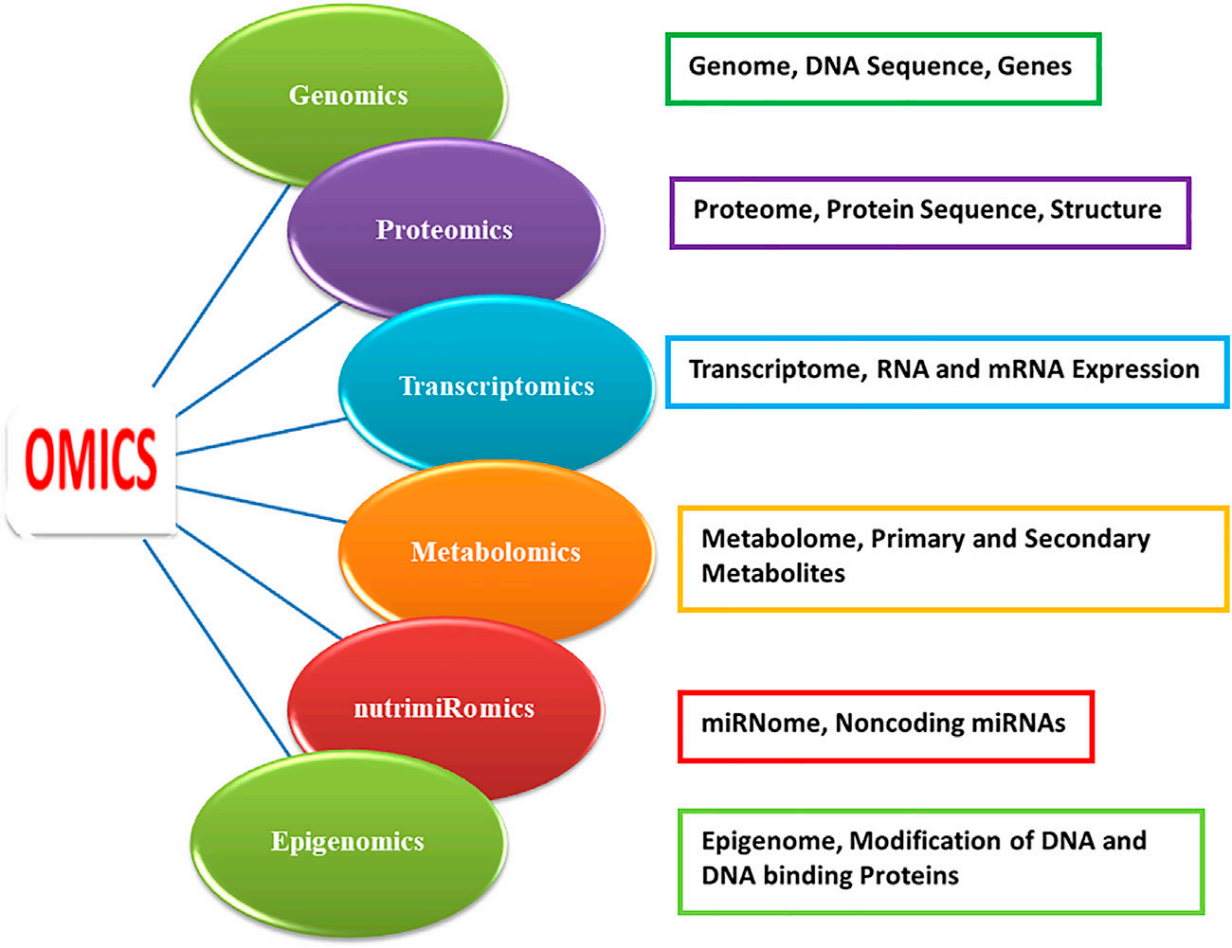
Omics branches with their targets.

The term “nutraceuticals” was coined by Dr. Stephen (1989). Nutraceuticals comprise “any nontoxic food extract supplement that has scientifically proven health benefits for both disease treatment and prevention” (DeFelice, 1995). Nutraceuticals combines the two disciplines of nutrition and pharmaceuticals (Figure 1). But, after the act of the dietary supplement health and education (1994) came into force, the designation of nutraceuticals stretched to include minerals, vitamins, herbs, extra-botanicals, amino acids, and dietary substances for usage as a diet supplement by humans (Stauffer, 1999). Instead of the nutraceutical term, the term dietary supplement is well-accepted globally and in the regulatory systems. About 470 nutraceutical and functional food products were accessible in 1999 with recognized fitness profits (Brower, 1999). Functional food or medicinal foods is a name entitled for edible foodstuffs with improved content of bioactive compounds by breeding, ecological influences, or genomic engineering and fortified foods that deliver health benefits, besides elementary nutrition. The term “functional food” was coined in Japan (1980) (Arihara 2014). Roberfroid (1999) defined functional foods as “products that have a relevant effect on well-being and health or result in reducing the risk of diseases.” However, an internationally accepted definition of functional food and nutraceuticals is not available (Arihara 2014; Télessy et al., 2019). Functional food is mainly referred to as “processed foods having disease-preventing and/or health-promoting benefits in addition to their nutritive value” (Arihara 2014 **)**. Functional foods cover nutraceuticals, pharma foods, probiotics, designer foods, medical foods, and vita foods (Arihara 2014). “Nutraceutical” is a food or food part that provides health benefits and encompasses disease prevention and treatment. Products as diverse as isolated nutrients, dietary supplements, and diets to “designer” foods, synthetic products, herbal products, and processed foods fall under the umbrella of nutraceuticals. Nutraceuticals are beyond food but subordinate to pharmaceuticals (Dudeja and Gupta 2017; Mohanty and Singhal, 2018; Télessy et al., 2019). Functional foods and nutraceuticals (FFNs) possess numerous health benefits. Besides essential nutrients, for example, fatty acids, carbohydrates, proteins, vitamins, and minerals, non-essential nutritional and bioactive food components such as folates, phenolics, polyamines, flavonoids, anthocyanin and nonflavonoid condensed tannins (ellagitannins), and carotenoids modulate various cellular processes (Abbasi et al., 2015; Kumar et al., 2018; Islam et al., 2021; Nayak et al., 2021). FFNs provide protection against disorders, for instance, cancer, obesity, high blood pressure, cardiovascular diseases (CVDs), gastrointestinal tract disorder, type II diabetes, inflammation, microbial, viral and parasitic infections, psychotic diseases, spasmodic disorders, ulcers, etc. (Abbasi et al., 2015; Kumar et al., 2018; Hamid and Hamid, 2019; Islam et al., 2021; Nayak et al., 2021; Salunkhe et al., 1983). Different omics platforms (Figure 1) will help in deepening our knowledge of food–body interactions, the influence of functional foods on consumption and the mechanism of their actions, safety issues, improvement of the nutritional value of staple foods, efficient use of nutrients, and characterization and development of new functional foods from traditional medicines and nutritional security (Kato et al., 2011; Abbott, 2014; Tian et al., 2016; Pazhamala et al., 2021). This review is an elaborate and informative type of review that describes the current status and efficient progress of various omics technologies in the arena of nutraceutical and functional foods. This review which discusses compiled data of multi-omics studies in nutraceutical and functional foods will enlighten our understanding of the potential of efficient foods and nutraceuticals for the betterment of human health.

## Genomics

Earlier, nutrition or FFN investigations were piloted principally for reviewing the significance of functional food or nutrient by its deficiency expressed as health-linked complications. Intermingled concepts of diet and health bonds were strongly recognized, though, only after the wealth of genomic information in form of three billion bases in every human cell was produced by Human Genome Project (HGP) in the year 2003, the nutritional research area developed to emphasize on the direct communication between nutrient and human genome (Venter et al., 2001; Austin, 2004).Single-nucleotide polymorphisms (SNPs) within human populations revealed variance in rejoinder to dietary nutrients (Subbiah, 2007). Therefore, nutritional genomics has developed due to the revolution of human genomics. Nutritional genomics consists of nutrigenetics and nutrigenomics:

### Nutrigenetics

Nutrigenetics estimates regulation of gene nutrition by presenting exactly how SNPs within persons influence their responses to nutritional constituents.

### Nutrigenomics

Alternatively, nutrigenomics scrutinizes nutrition and regulation of gene expression by indicating how dietary nutrients interconnect with human genomic sequences and change their gene expression and gene transcripts. It provides authorization for nutrition–gene regulation owing to advanced omics approaches (Kato, 2008). Nutrigenomics also denoted as Nutri-Omics or nutritional genomics is a sub-discipline of food and nutrition research *via* the application of various highly effective high-throughput genomics (McGuire et al., 2020; Müller and Kersten, 2003) and omics-based approaches including transcriptomics, proteomics, epigenomics, nutrimiRomics, metabolomics, and bioinformatics (Pazhamala et al., 2021) (Figure 1). Cominetti et al. (2017) referred to nutrigenomics as the investigation of communications between food nutrient ingredients and human genome and evaluation of genomic expression and metabolic functions after nourishing. The prompt progression of technologies, for example, high-throughput sequencing of the genome (DNA) and transcriptome (RNA), proteome sequencing by mass spectrometry (MS), and upgraded outcome of DNA microarrays has contributed greatly to expanded applicability of omics platforms in the area of food science and directed field of nutri-omics to the right direction (Fu et al., 2010; Gehlenborg et al., 2010). Today different nutri-omics platforms are prerequisites to gain deeper insights into and knowledge of the influence of dietary food components on humans along with the mechanism of their actions. The overall goal of nutrigenomics appreciates in what manner nutrition impacts cell metabolic pathways and homeostatic regulation. Furthermore, how this regulation interrupts the initial stage of a lifestyle diet–linked ailment depends on the genotypes of the individual (Müller and Kersten, 2003). The bond between nutrition and human health is essentially affected by the interaction between the nutrient and genes. The functional sensitivity of genes to nutrients delivers the central foundation of nutrigenomics. Both while in the uterus of the pregnant mother and through the primary years of life, under and over-nourished mother–child components imprint gene modifications causing chronic metabolic complications in the future life of humans (Chávez and Muñoz de Chávez, 2003).

## Transcriptomics

Among all omics platforms, transcriptomics appears to be the utmost efficacious and extensively used technology in the field of nutrigenomic studies due to its efficiency and high data representation (Kato, 2008). Transcriptome includes the whole mRNA or transcript complements transcribed or expressed from genes. So, transcriptomics is a potent platform for expression profiling of various genes in a genome (Lowe et al., 2017). Diverse bioactive constituents of food can affect the gene expression in primary to changed cellular biological processes along with cell metabolism, cell proliferation, and tissue differentiation, and the death of the cell and their imbalance may cause syndromes such as diabetes or cancer. Owing to that, gene expression interrogation at the genome level influenced by dietary functional nutrients is predominantly significant in nutrigenomics studies. Transcriptomics for investigating the influence of food components on the expression of genes has been adopted in diverse investigational paradigms together with animal cell cultures, animal models, and human beings. With transcriptomics technology, clinical trials on obese humans with dietary intervention have been carried out to check the influence of energy-restricted diets on the expression of genes present in adipose tissue (ClÉment et al., 2004; Dahlman et al., 2005). The human transcriptomic study assessed duodenal mucosa for the gene expression profiles of Lactobacillus G Gon (DiCaro et al., 2005). The transcriptomic investigations for understanding the variations in global gene expression due to various dietary interventions, for example, deficiency of nutrients, fasting, ingestion of disproportionate nutrients, and specific food factors have been performed (Endo et al., 2002; Matsuzaki et al., 2005; Kamei et al., 2010; Nakai et al., 2008; Saito et al., 2010; Ohta et al., 2006; Tachibana et al., 2005). The transcriptomic investigation of rat liver that was subjected to mild caloric restriction (5–30 percent lesser amount of food for 1 week or 1 month) (Saito et al., 2010) was carried out to examine food functionality by discriminating direct consequences of food nutrients and secondary effects produced by alterations in food ingestion behavior. The cyp4a14 gene expression revealed restriction level–dependent changes, so it can be used as a biomarker for the beneficial effects of functional food nutrients on energy metabolism. Microarray technology allows simultaneous quantification of thousands of mRNA before and after exposure to bioactive dietary components (Kato, 2008; Garosi et al., 2005). The DNA microarray tool has been used in *in vivo* conditions for documentation of cellular responses to food components and their cellular targets at molecular levels in various investigations, for instance, green tea catechins (McLoughlin et al., 2004; Vittal et al., 2004), vitamin D and vitamin E (Johnson and Manor, 2004; Lin et al., 2002), polyunsaturated fatty acids (Lapillonne et al., 2004; Kitajka et al., 2004; Narayanan et al., 2003), soy isoflavones (Herzog et al., 2004), quercetin (Murtaza et al., 2006), anthocyanins (Tsuda et al., 2006), arginine (Leong et al., 2006) and hypoallergenic wheat flour (Narasaka et al., 2006). Some other transcriptome expression studies involving the use of microarray are listed in Table 1. Apart from expression studies, transcriptomics has also been practiced to evaluate the safety of food (Kato, 2008). The swift accretion of nutri-transcriptomic microarray data stimulated the formation of an integrated open-source web-based database which guarantees efficient organization, storage, and investigation of the huge volume of microarray data produced from all nutri-transcriptomic research studies (Saito et al., 2005).

**TABLE 1:** Studies of transcriptome expression analyses by using DNA microarray technology.

Organism	Experiment	Consequences	Reference/s
MCF-7 breast cancer cells	Influence of genistein on gene expression at global levels at physiologic (1 or 5 mu M) and pharmacologic (25 mu M) concentrations	Genistein altered the expression of genes of various pathways, plus estrogen- and p53-mediated pathways. At physiologic concentration (1 or 5 µM), genistein elicited an elevated expression and mitogenic activity, while at the pharmacologic concentration (25 µM), genistein escalated apoptosis, reduced proliferation, and total cell number	Lavigne et al. (2008)
Rat model of an alcohol-induced fatty liver	Analysis of hepatic gene expression	The five genes (β-glucuronidase, UDP-glycosyl transferase 1, UDP glucose dehydrogenase, apoC-III, gonadotropin-releasing hormone receptor) involved in immune response, signal transduction, transcription, and protein and amino acid metabolism were controlled by chronic ethanol intake	Park et al. (2008)
Blood cell RNA of eight healthy men prior and post 2 h diet ingestion	Effect of high-carbohydrate (HC) or high-protein (HP) breakfast on the transcriptome of human blood cells	Genes (317) for HC breakfast and genes (919) for HP breakfast showed differential expression. HC breakfast ingestion showed differential gene expression of mainly glycogen metabolism and HP breakfast showed differential expression of protein synthesis genes	van Erk et al. (2006)
Lymphocytes from 30 post-menopausal women	Effects of dietary soy isoflavones on changes in expression of genes	Isoflavones had a robust effect on some putative estrogen-responsive genes in equol producers than non-producers due to enlarged cell differentiation, cAMP signaling, G-protein–coupled protein metabolism and steroid hormone receptor activity	Niculescu et al. (2007)
Subcutaneous adipose tissue (SAT) in 47 persons with metabolic syndrome	Gene expression after consumption of two carbohydrate modifications (rye–pasta diet with low postprandial insulin response and oat–wheat–potato diet with high postprandial insulin response)	The rye–pasta diet downregulated 71 genes related to insulin signaling and apoptosis. The oat–wheat–potato diet upregulated 62 genes connected to cytokine-chemokine-mediated immunity, stress, and interleukin pathway	Kallio et al. (2007)
Adipose tissue from 131 moderately overweight men	Identification of molecular pathways responsive to caloric restriction and dietary composition	Above 1,000 transcripts showed downregulated expression after acute weight loss. The expression of stearoyl-coenzyme A desaturase (SCD) in adipose tissue is autonomously controlled by weight loss and by ingestion of carbohydrates and saturated fat. The expression of SCD and diacylglycerol transferase 2 (DGAT2) may be implicated in the dietary regulation of triacylglycerol metabolism	Mangravite et al. (2007)
Rectal mucosa in randomized double-blind crossover trial on 19 healthy volunteers	Effect of daily intake of low-digestible and prebiotic isomalt and digestible sucrose on gene expression for 4 weeks of feeding	No influence on gene expression in lining rectal mucosa after dietary intervention while gene expression of the rectal mucosa can be measured in biopsy material	Schauber et al. (2006)

## Proteomics

Proteomics involves high-throughput analysis of proteomes in cells, tissues, or biological fluids which are being expressed by the genome (Thongboonkerd, 2007; Husi and Albalat, 2014; Hixson et al., 2017) and also facilitates novel protein discovery. The proteome is the complete complement of proteins expressed from a set of specific genes in any biological organism at a given point of time and specific environment (Trayhurn, 2000; Hixson et al., 2017). The proteome is dynamic, continuously changes in line with cell type and cell functional state, and extra complex than the genome (Thongboonkerd, 2007; Hixson et al., 2017). From the genome of humans, nearly 25,000 functional genes are encoded, whereas the proteome includes about 250,000 proteins because of alternative splicing and posttranslational modifications (Kussmann and Affolter, 2006). The multifaceted proteome can be evaluated by proteomics tools. Proteomics covers protein investigation by protein separation, protein quantification, and protein identification (Kussmann et al., 2008) and has promptly proceeded from gel-based techniques such as 2-DE (two-dimensional) gel electrophoresis to technologies such as mass spectrometry (MS) by evaporation of peptides and proteins by MALDI (matrix-assisted laser desorption/ionization) and ESI (electrospray ionization), multiple reaction monitoring, and multiplexed immunoassays (Swatton et al., 2004; de Roos and McArdle, 2008; Zhang et al., 2014). Nutritional proteomics provides knowledge about the complex interaction of nutrition-protein regulation, identification of new biomarkers for dietary status, and develops novel stratagems for diet-related avoidance and interference of ailments (Ovesná et al., 2008). Nutritional proteomics, which is an essential fragment of nutrigenomics, studies the influences of functional food nutrients on the expression of proteins and delivers prospectives for differentiating biomarkers that show sensitivity to dietary interventions (Fuchs et al., 2005a). Limited nutritional proteomics analysis in humans involved cell culture (colon cancer cell and endothelial cell) studies which confirmed the effects of food components (butyrate, flavonoid, and genistein) on protein profiles and identified the cellular molecular target proteins of components of the diet (Tan et al., 2002; Herzog et al., 2004; Wenzel et al., 2004; Fuchs et al., 2005b; Fuchs et al. 2005c; Fuchs et al. 2005d). Exponentially cumulative publications on proteomics in nutritional research showed its prospective (de Roos et al., 2005; Breikers et al., 2006; Griffiths and Grant, 2006; Kim et al., 2006). Proteomics of liver tissues of two mice strains that were susceptible and resistant to atherosclerosis led to the identification of 30 differentially expressed proteins of oxidative stress and lipid metabolism which were significantly altered in response to an atherogenic diet proposing that the identified proteins add to variances in atherogenesis susceptibility (Park et al., 2004). With the protein microarray, the cholesterol diet–induced expression of proteins revealed an altered pattern of phosphorylation (Puskas et al., 2006). The 2D gel and MALDI-TOF-MS proteomic analysis techniques identified and characterized novel prospective allergens from transgenic soybean and nontransgenic soybean (Batista et al., 2007), allergens from wheat flour, and it was discovered that the nine glutenin subunits are utmost principal IgE-binding antigens (Akagawa et al., 2007) and 15 host defense proteins were identified (Smolenski et al., 2007). After intervention with flaxseed diet in seven humans, the PBMC—peripheral blood mononuclear cell proteome showed significant influence on steady-state levels of sixteen proteins and improved peroxiredoxin and decreased long-chain fatty acid beta-oxidation multienzyme complex and glycoprotein IIIa/II levels (Fuchs et al., 2007). There is a public 2-DE database for proteome of human PBMCs (peripheral blood mononuclear cells), with the potential to examine the proteomics alterations concomitant to interventions of diet or drugs (Vergara et al., 2008). Comparative proteomics between rat livers subjected to 30% food restriction and control rats led to identification of nine proteins showing upregulation and nine proteins with downregulation. The 10% food restriction caused nine upregulated and two downregulated proteins with the 2-DE technique. The prohibitin which regulates longevity was also upregulated (Artal-Sanz and Tavernarakis, 2009) and can prove to be a highly promising and efficient proteomic biomarker for positive results of food components (Kussmann et al., 2010a). In the food and nutrition proteomics research approach, the influence of trivial caloric restriction was also investigated (Takahashi et al., 2011). The rats were fed on a GSE—grape seed extract–supplemented diet. Their homogenates of brains when subjected to proteomics investigation lead to the identification of thirteen candidate proteins (Deshane et al., 2004). Some of these proteins were under regulation by supplementation of grape seed extract in opposed order from earlier studies for the identified proteins in mouse models of Alzheimer’s neurodegeneration syndrome signifying their modulation by GSE as a neuroprotective agent (Deshane et al., 2004). With mass spectrometry (MALDI-TOF MS) serum protein B-chain of α2-HS glycoprotein diet–related biomarkers with a role in insulin resistance and immune function were isolated from human subjects fed on a cruciferous-supplemented diet in contrast to control human subjects (Mitchell et al., 2005). Mingling of transcriptomics with proteomics leads to the identification of enzymes and transporters involved in fatty acid metabolism, sequestration, and transcriptional regulation in zinc-deficient models. The hepatic lipid accretion pathways were designed (tom Dieck et al., 2005).

## Metabolomics

Nutritional metabolomics is a unique modern omics know-how in research (Rochfort, 2005) involving the investigation of global metabolite profiles in any organism in precise ecological scenarios. Metabolomics provides the perception of biochemical deviations after the intervention of diet and impacts safety issues of GMO—genetically modified food (Dixon et al., 2006). The metabolome comprises a complete set of metabolites biosynthesized in any biological organism, whereas the metabolites are final products of biological reactions of metabolism, signifying the genome–environ interaction (Rochfort, 2005). Beyond 10,000 categories of key metabolites being existent in animals, the figure for proteins is assumed to drive beyond 100,000. This metabolite feature probably results in extra-broad features of metabolomics compared to proteomics. Metabolite analysis is challenging and frequently needs the usage of high-level techniques owing to much greater diversity and abundance of metabolites than mRNA transcripts and proteins. In spite of these technical hitches, metabolomics is a potent technique in neutraceuticals and food nutrition (Oresic, 2009; Zivkovic and German, 2009) and uses NMR—nuclear magnetic resonance, MS—mass spectrometry (gas chromatography (GC)-MS and liquid chromatography (LC)-MS and capillary electrophoresis (CE)-MS (Prakash et al., 2018; Kumar et al., 2019). NMR and MS in conjunction with potent bioinformatics podiums significantly boost metabolomics in the investigation of nutrition (German et al., 2003; Trujillo et al., 2006; Hall et al., 2008). The nutrition metabolomics study performed in five fit premenopausal women under controlled state of affairs determined the biochemical changes subsequent to clear intervention of diet with soy isoflavones and showed that soy-induced isoflavones brought modifications in plasma components such as plasma lipoprotein, amino acid, and carbohydrates with a role in energy metabolism (Solanky et al., 2003). Other examples include deducing the metabolic consequences of vitamin E supplements in *in vitro* conditions in a mouse model of motor neuro-degeneration, evaluation of human biological reaction to various diets, for example., chamomile tea or vegan, low- and high-meat diets, the characterization of the variations in metabolic profile attributable to dissimilar populations such as Chinese, American, and Japanese or Swedish and British inhabitants (Dumas et al., 2006; Griffin et al., 2002; Lenz et al., 2004; Wang et al., 2005; Stella et al., 2006). Metabolite profiles of chamomile tea consumption in humans estimated with chemometric methods and ^1^HNMR spectroscopy exhibited a high degree of variation with clear discrepancy in excretion of urine, signifying augmented hippurate and glycine and reduced creatinine levels (Wang et al., 2005). The influence of green tea ingestion in humans showed a robust upsurge in intermediates of citric acid cycle urinary metabolites suggestive of the influence of flavanols present in green tea on oxidative energy metabolism (van Dorsten et al., 2006). The metabolic response of rats fed with whole-grain flour and refined wheat flour showed that some intermediates of the TCA(tri carboxylic acid) cycle, aromatic amino acids, and hippurate significantly escalated in the urine of rats nourished on whole-grain flour (Fardet et al., 2007). The proton NMR-based metabolomics approach investigated and identified the overall biochemical consequences of short-term high consumption of animal milk protein or meat protein on 8-year-old boys and found that milk diet augmented excretion of hippurate and marginally altered serum lipid metabolic profile, while the meat diet amplified excretion of histidine, creatine, and urea in urine with no influence on the serum profile (Bertram et al., 2007). Nutimetabolomics contributed to the revolution of omics. Human food constituent–induced changes in nutritional metabolic profiles are not well-understood, so the global picture of human metabolism is not imaginable. Technological advancements can overcome future challenges of using metabolomics in nutrition research (Ovesná et al., 2008).

## Epigenomics

Epigenomics is the investigation of the epigenome–plastic variations at the level of epigenetics, for example, cytosine DNA methylation and histone modifications devoid of variations in DNA nucleotide sequence and small RNA–mediated methylation (Kussmann et al., 2010b; Yadav et al., 2018; Lloyd and Lister, 2022). Epigenetics modifications modulate gene expression at respective locations (Yadav et al., 2018). The pregnant mother’s nutrition during the development of the fetus influences the acquired predisposition to lifestyle-linked disorders such as hypertension, obesity, diabetes, cardiovascular diseases, etc in future (Lusis et al., 2008) on the basis of epigenetic alterations. NGS—next-generation sequencing and genome-wide analysis technologies may throw light on the epigenomic modification in nutritional omics (Hawkins et al., 2010). Effect of nutrition on initial life stages, for example, fetal, suckling, and growing age on health in advanced stages of life may be allocated by chromatin modifications in future.

## Nutri miRomics

A new emergent area of omics platforms in functional foods is associated with RNA transcripts not translating into proteins. MicroRNAs (miRNAs) are small, single-stranded, endogenously encoded, 18–25 nucleotide conserved, non-coding RNAs that regulate expression of the protein-coding genes by PTGS—posttranscriptional gene silencing (Pandita, 2018a) and possess roles in nutrient homeostasis, hormone homeostasis, signaling pathways, immune response, human disorders, etc. (Pandita, 2018b; Pandita and Wani, 2019; Pandita 2021). A non-coding gene previously referred to as “JUNK” under the spotlight as “just unrevealed new-fangled know-how” of future treasure veiled in genomes that produce functional RNA molecules for internal signals and hold potential in medicine at personalized levels (Pandita and Pandita, 2016). Since the accruing confirmations support the significance of miRNA in disease development (Pandita, 2018a) and health maintenance, the know-how on miRNA eminence is, indeed, indispensable for understanding the interaction between functional food constituents and the human body. Nutrient such as phosphate helps in the processes of replication of DNA, development of phospholipid bilayer, and biosynthesis of ATP molecules, whereas the deficiency of P causes upregulation and downregulation of miRNAs. Global microRNA investigation can be performed by high-throughput tools of microRNA sequencing and microRNA microarray (Pandita, 2019). RNAi triggered by short double-stranded small interfering RNA (siRNA) has been used in commercial crops such as cassava plants deprived of cyanogenic linamarin, tomato plants with few allergens, dietary antioxidant–rich fortified tomatoes, Flavr Savr tomato, and ring spot–resistant cultivars of papaya plant (Pandita, 2018b). Nutrimiromics investigates the influence of food on gene expression modifications because of microRNA epigenetics affecting the jeopardy of chronic disease development. Nutrimiromics comprehends the association between food components and the reaction of microRNAs in particular body parts. Nutritional supplements are prospectively prized army to fight against obesity—the imbalance between food consumption and energy outflow (Downs et al., 2005; Lau et al., 2008; Li et al., 2008). Whole-genome screening approaches enabled the identification of coding genes sensitive to nutritional supplements. The hypotheses can be formulated by explaining the mode of action of the supplement under interrogation (Roy et al., 2004; Roy et al., 2007). Expression profiling has provided innumerable new adipose tissue genes for the regulation of obesity. Assimilating expression patterns with genome-wide linkage and association maps can recognize novel obesity susceptible genes (Dahlman and Arner, 2007). MicroRNAs induced during adipogenesis fast-track development of fat cells and show downregulation in obesity. Ectopic expressions of microRNA-103 or microRNA-143 present in preadipocytes speed up adipogenesis, proving that miRNA plays a crucial function in obesity (Xie et al., 2009). Angiogenesis helps in adipose tissue growth and is controlled by miRNAs (Sen et al., 2009). Nutritional supplements against obesity need to check for adipose tissue miRNA regulation for speeding up the catabolism of fat at cellular and tissue levels. Nutri miRomics represents a potent tool and will probably emerge as a key pilot of forthcoming nutritional supplement trade. Resolvins—DHA and EPA metabolites develop due to injury of tissues that back inflammation homeostasis by NF-kB downregulation (Zárate et al., 2017). The microRNA-21, microRNA-146b, microRNA-142 family, microRNA-203, microRNA-208a, microRNA-219, and microRNA-302d show temporal and differential expression in tissue exudates and resolvin D1 biosynthesized in resolution controls microRNA-21, microRNA-146b, microRNA-208a, and microRNA-219 (Recchiuti, et al., 2011). In HepG2 cells, EGCG downregulates microRNA-30b, microRNA -453, microRNA-520-e, microRNA-629, and microRNA-608 which are involved in glycolysis and gluconeogenesis, inflammatory pathways, insulin signaling pathway, oxidative phosphorylation, peroxisome proliferator–activated receptors (PPARs), signaling pathway, and glutathione metabolism (Arola-Arnal and Bladé, 2011). EGCG upregulated the miR-let-7b in melanoma cell lines and metastatic melanoma tumors and suppresses melanoma tumor growth by activation of the intercellular signaling pathway, cAMP/protein kinase A (PKA)/protein phosphatase 2A (PP2A) (Yamada et al., 2016). From the blood of mice nourished on 10 g of rapeseed bee pollen/kg of body weight, 132 plant-derived microRNAs (miR-166a and miR-159a were profuse) were identified (Chen et al., 2016). In the plasma samples of four healthy adult humans who ingested 102 g of broccoli, no variation was detected in broccoli-derived microRNA-824 and microRNA-167a after 4 h of broccoli ingestion (Baier et al., 2014). Table 2 lists some other studies where the principal miRNAs are modulated by the respective nutrient and bioactive compounds.

**TABLE 2:** Studies of miRNAs modulated by respective nutrient and bioactive compounds.

Experiment	Consequences	Reference/s
THP-1 cells were incubated with Resveratrol (30 or 50 µM) for 14 h	Anti-inflammatory miR-663 up-regulated, which targets two AP-1 factors (Jun B and Jun D) reducing AP-1 activity. Pro-inflammatory miR-155 down-regulated and miR-663 up-regulated	Tili et al. (2010)
THP-1 cells were incubated with resveratrol (25, 50, 100, and 200 mM) for 48 h	Upregulation of miR-Let7A in treated cells compared to non-treated cells. Resveratrol and/ormiR-Let7A target mRNA of TNF-α and IL-6 and amplified IL-10 after stimulation of cells with LPS.	Song et al. (2016)
Human glioblastoma (U251) cells were treated with 10 or 50 µM resveratrol for 12 h to check the effect of resveratrol on the expression of miR-21	The phenolic compound resveratrol inhibited and reduced expression of pro-inflammatory miR-21 in-turn causing a reduction in the activity of IkB phosphorylation and NF-kB	Li et al. (2013)
Macrophages (RAW 264.7) incubated with concentrations of resveratrol, hydroxytyrosol, and oleuropein compatible with plasma physiological concentrations (5 and 10 µM)	Resveratrol and hydroxytyrosol (at 10 µM) downregulated miR-146a which targets the nuclear factor (erythroid-derived 2)–like 2 (Nfr2) transcription factor with a role in the inhibition of pro-inflammatory mediators. Nfr2 was positively modulated by resveratrol and hydroxytyrosol after macrophage stimulation with LPS.	Bigagli et al. (2017)
Randomized placebo-controlled study on 35 type-2 diabetic and hypertensive men who consumed capsules with placebo (maltodextrin), grape extract (devoid of resveratrol) (GE), and grape extract with over 8 mg of resveratrol (GE-RES) during 1 year	In the group supplemented with GE-RES, miR-21, miR -181b, miR-663, and miR -30c2 were upregulated and miR-155 and miR-34a were downregulated as compared to the control group	Tomé-Carneiro et al. (2013)
Treatment of human myocytes (L6 GLUT4myc) with palmitic acid	MiR-29a levels enhanced causing posttranscriptional inhibition of insulin receptor substrate (IRS)-1 and reducing protein concentration	Yang et al. (2014)
EPA- and DHA-induced stimulation of macrophages (RAW 264.7) and epithelial (TIME) cells with LPS and pro-inflammatory cytokines (IL-1β, TNF-α, and IFN-γ) and the treatment of these cells with DHA (C22:6n3) or arachidonic acid (AA, C20:4n6)	The anti-inflammatory action of PUFAs was mediated by downregulation of miR-146a, miR-146b, miR-21, miR-125a, and miR-155 linked with pro-inflammatory response triggered by NF-kB signaling	Roessler et al. (2017)
The treatment of mouse cardiomyocytes (HL-1 cells) with palmitic acid	Stimulation of miR-27b expression signifying an enhanced vulnerability to atrial arrhythmia	Takahashi et al. (2016)
Leukotriene B4 synthesized from arachidonic acid in mice macrophages	Stimulated the inflammatory response by increasing MyD88 *via* upregulation of miR-155 and miR -146b, which are responsible for SOCS-1 mRNA degradation and MyD88 inhibition	Wang et al. (2014)
Treatment of hepatocytes with oleic acid	Reduced PTEN expression by upregulating miR-21 *via* a direct effect of NF-kB p65 on the miR-21 promoter	Vinciguerra et al. (2009)
Humans(30) consumed 30 g/day of almonds and nut sources of polyunsaturated fatty acids (PUFA) for 8 weeks	miR-328, miR-330-3p, miR-221, and miR-125a-5p had their expressions reduced, while miR-192, miR-486-5p, miR-19b, miR-106a, miR-130b, miR-18a, and miR-769-5p displayed increased levels after the intervention. miR-221 and miR-130b were associated with positive variations in plasma protein C-reactive (PCR) levels	Ortega et al. (2015)
Treatment of breast cancer cells with curcumin	The upregulation of miR-181b is related to a down-modulation of pro-inflammatory cytokines CXCL1 and -2, causing an inhibitory effect on the metastatic process of these cells	Kronski et al. (2014)
Treatment of breast stromal fibroblast with curcumin	Tumor suppressor p16INK4A protein inhibits carcinogenic effects of cells by repressing IL-6 expression and secretion. This process is mediated by miR-146b-5p which inhibits the expression of cytokines at a specific sequence at IL-6 3′UTR. After curcumin treatment, p16INK4A and miR-146b-5p levels increase and suppress IL-6	Al-Ansari and Aboussekhra, (2015)
Female mice consumed quercetin-enriched diets (2 mg/g), compared to controls with the control diet	Hepatic levels of miR-125b (negatively regulates inflammation) and miR-122 (regulates lipid homeostasis) showed upregulation in female mice fed on quercetin-enriched diets.	Boesch-Saadatmandi et al. (2012)
A harvested heart of a rat model with selenium deficiency	MiR-374, miR-16, miR-199a-5p, miR-195, and miR-30e involved in cell differentiation, signal transduction, and stress-response were upregulated >5-fold in the deficiency group than the selenium-supplemented group. The miR-3571, miR-675, and miR-450a were downregulated	Xing et al. (2015)
Humans consumed a zinc depletion dietary regimen	Dietary zinc depletion–responsive 20 miRNAs were shown and reversed by succeeding zinc repletion. Highly downregulated miR-204 and miR-296-5p suppress oncogene expression	Ryu et al. (2011)

## Concluding Remarks

Plants are the picture-perfect cradle of FFNs owing to the presence of thousands of naturally valuable bioactive metabolites and phytochemical food compounds which can be augmented by genetic improvement. The biotechnological omics tool goals in nutrigenomics research range widely from identification, isolation, and innovation of new functionality, manipulations, revelation of the mode of action and security concerns of specific health-stimulating metabolites and phyto-compounds or their combination for designing and developing FFNs which can prove as a key stratagem in complementary medicine. The ultimatum of omics-based approaches will endure for growth and development in food and nutrition sciences. The extensive investigation of one specific omics approach will expand the understanding of the communication between essential food constituents and the human body. The permutation and unification of various omics data will arrange for supplementary concrete statistics on what takes place within the body in reaction to food consumption. Furthermore, the integration of various omics approaches or the integrated omics may intensify the significance of omics research.
